# Evaluierung der NEF-Fehl- und Übergabeeinsätze im Raum Innsbruck: Retrospektive Auswertung der NEF-Stornierungen und Übergabeeinsätze vom Notarzt an den Rettungsdienst in den Jahren 2017 und 2018

**DOI:** 10.1007/s00101-021-01046-y

**Published:** 2021-10-13

**Authors:** Teresa Troppmair, J. Egger, A. Krösbacher, A. Zanvettor, A. Schinnerl, A. Neumayr, M. Baubin

**Affiliations:** Universitätsklinik für Anästhesie und Intensivmedizin, Anichstraße 35, 6020 Innsbruck, Österreich

**Keywords:** Notarztstornierung, Übergabeeinsatz, Notarztindikationskatalog, Dokumentation, Qualitätsmanagement, Emergency physician cancellation, Patient handovers, Quality control, Documentation, Indication checklist

## Abstract

**Hintergrund:**

Die Qualität eines Rettungssystems zeichnet sich auch durch den effizienten Einsatz seiner personellen und Fahrzeugressourcen aus. So können im berechtigten Fall Stornierungen des anfahrenden Notarztes durch den Rettungsdienst (RD) ebenso sinnvoll sein wie Übergaben des stabilen Patienten an den RD. Aufgrund der hohen Zahlen solcher Storno- und Übergabeeinsätze evaluiert diese Studie diese Entscheidungen retrospektiv und zeigt evtl. Auffälligkeiten auf. Studienkollektiv waren die 10.278 Notarztalarmierungen der beiden Notarzteinsatzfahrzeuge (NEF) Innsbruck Stadt (städtisch) und Telfs (ländlich) der Jahre 2017 und 2018.

**Methode:**

Der Patientenzustand sowie die Rettungsdienstdokumentation wurden beurteilt und die Notarztindikation retrospektiv anhand der klinischen Aufnahmediagnosen im Abgleich mit dem Notarztindikationskataloges der Deutschen Bundesärztekammer (NIKDBÄK) anhand vorgegebener Kriterien wie eingegebener Vitalparameter und/oder des Notfallgeschehens bewertet.

**Ergebnisse:**

Im zweijährigen Studienzeitraum ergaben sich 2470 relevante Datensätze, davon 1190 Storno- und 1280 Übergabeeinsätze mit gesamt 210 Einsätzen (8,5 %) mit Notarztindikation laut NIKDBÄK. Am NEF Innsbruck fanden mehr Stornierungen statt, und es kam zu mehr Storno- als Übergabeeinsätzen, umgekehrt dazu am NEF Telfs zu mehr Übergabe- als Stornoeinsätzen. An Wochenenden fanden nachts weniger Storno- und Übergabeeinsätze statt. In 284 Protokollen bei Stornierungen (23,9 %) und 339 Protokollen bei Übergaben (26,5 %) war die Dokumentation der Sanitäterprotokolle unvollständig. Patienten mit gegebener Notarztindikation laut NIKDBÄK mussten länger stationär behandelt werden. 35 Patienten nach Storno- (2,9 %) und 35 Patienten nach Übergabeeinsätzen (2,7 %) mussten auf einer Intensivstation aufgenommen werden. Bei den Intensivbehandlungen wurde bei 20 Patienten (1,7 % der Stornoeinsätze) nach einem Stornoeinsatz eine kritische Aufnahmediagnose festgestellt bzw. bei 24 Patienten (1,9 % der Übergabeeinsätze) nach einem Übergabeeinsatz. Bei 40 (3,1 %) Übergabeeinsätzen vom Notarzt an den Rettungsdienst kam es innerhalb von 10 min nach Eintreffen des Notarztes zu einem Folgeeinsatz.

**Schlussfolgerung:**

Die Einführung eines eigenen standardisierten Notfallindikationskataloges für Österreich erscheint als Vorgabe für Leitstellen und Rettungsdienstpersonal sinnvoll. Storno- und Übergabeentscheidungen müssen sorgsam getroffen werden und sollten QM-gesichert evaluiert werden. Der Dokumentationspflicht im Rettungswesen muss vermehrte Aufmerksamkeit gewidmet werden. Durch intensivere Aus- und Fortbildungen sowie Diagnosefeedbacks könnte die Anzahl an unberechtigten bzw. risikobehafteten Storno‑/Übergabeeinsätzen vermindert werden.

**Zusatzmaterial online:**

Die Online-Version dieses Beitrags (10.1007/s00101-021-01046-y) enthält eine Tabelle mit der Auflistung der Indikationen.

## Abstract (en)

**Background:**

Human and vehicle resource management indicates a good emergency medical system (EMS). Frequently, an emergency medical technician (EMT) is the first responder to the emergency, which negates the necessity for an emergency physician (EP) and is just as sensible as handing over a stable patient to the EMT for transport to the hospital. The Austrian EMS is utilized by EMTs, in cases of potential life-threatening emergencies the dispatch center dispatches an additional team with an on-board EP. During the years 2017–2018 nearly every fifth EP mission in Innsbruck (including surrounding areas) ended in a cancellation. The numbers of patient handovers from EP to EMT are slightly lower with mission cancellations resulting in every fourth patient. Therefore, due to the high number of cancellations and handovers evaluated in this study, the findings suggest that there is a potential need to re-evaluate procedures. The re-evaluation of these procedures could determine whether these cancellations/handovers were justified or if an over hasty decision making was at fault. All cases considered in this study were from the Innsbruck and Telfs EP bases between 1 January 2017 and 13 December 2018.

**Methods:**

Out of a total of 96,908 emergency dispatches, there were 2470 cancellation/handover occurrences. These occurrences consisted of 1190 cancellations and 1280 patient handovers from the EP to the EMT. Patients who were transferred to the University Hospital Innsbruck were included in these figures. The protocols of the emergency dispatches have been filtered from the so-called CarPC. They have subsequently been grouped into cancellation and handover categories. The clinical diagnoses of the patients with inpatient treatment were evaluated from the hospital information system (KIS) of the University Hospital Innsbruck. This was done with the help of the so-called emergency physician indications catalogue of the German Medical Council. The diagnosis was documented in the hospital information system. The emergency protocols from the EMTs were also evaluated retrospectively. The Innsbruck based EP patients are hospitalized in the Innsbruck Hospital due their geographical position. When there is no need for a specific intervention the patients of the EPs based in Telfs are transferred to a local hospital. When a specific intervention is necessary, patient care must be provided by the University Hospital Innsbruck. Due to the privacy practices of the Innsbruck Medical University “vote of ethics” only the data of patients transferred to the Innsbruck Clinic can be evaluated. The information provided from the EPs based in Innsbruck was exclusively from the University Hospital Innsbruck’s anesthesiologists. The physicians from the Telfs EP base are of mixed medical specialities. All of them, however, have an emergency medical physician diploma, in addition to the *ius practicandi*. Lastly, there are no EPs in Innsbruck or Telfs, who have any special obligations during their duty.

**Results:**

The results show that in 210 cases (8.5%) the indications for the EP, based on the emergency physician indications catalogue of the German Medical Council were given. Also, 8.7% of all cancellations and 8.4% of patient handovers were not justified. Patients with emergency indications had a longer hospitalization. The EP base EMS Innsbruck had more cancellations than the EP base EMS Telfs. The EMS Innsbruck also had more cancellations than patient handovers. Conversely, the EMS Telfs had more patient handovers than cancellations. On the weekends between 6:00 pm and 6:00 am there were less cancellations and handovers from both EP bases. The documentation from the EMT protocols was incomplete in 284 cancellations (23.9% of the cancellations) and 339 handovers (26.5% of the handovers), 35 patients after cancellations (2.9%), 35 patients after handovers (2.7%) needed intensive care treatment, 20 patients after cancellations (1.7% of all cancellations), and 24 patients after handovers (1.9% of all handovers) who needed intensive care treatment had a critical diagnosis. In 40 cases of patient handovers, the EP was alerted to another emergency follow-up within 10 min.

**Conclusion:**

In Austria, the introduction of a standardized emergency indication checklist might help dispatch centers to provide a more accurate dispatch as well as all EMS team members. Furthermore, a better traceability system (according to EP cancellations and patient handovers from the EP to the EMT) could be achieved. The documentation requirements should be more precise by all members of the EMT staff, not only for the legal aspects but also for improving the overall management quality. Intense education and training as well as diagnosis feedbacks could help to reduce the number of risky cancellations/patient handovers.

Die Qualität eines Rettungssystems zeichnet sich auch durch den effizienten Einsatz seiner personellen und Fahrzeugressourcen aus. So können im berechtigten Fall Stornierungen des anfahrenden Notarztes (NA) durch den Rettungsdienst (RD) ebenso sinnvoll sein wie Übergaben des stabilen Patienten an den RD. Notfallpatienten müssen darauf vertrauen können, dass im Rettungsdienst bestausgebildetes und erfahrenes Personal die Notfallversorgung durchführt. Die 2‑stufigen Notarzt- und Rettungsdienstsysteme im deutschsprachigen Raum müssen einer effizienten Steuerung und regelmäßigen Evaluierung unterliegen.

Im angloamerikanischen Raum führen „paramedics“ notfallmedizinische Erstversorgungen durch. Notärzte können nur bei sehr speziellen Indikationen angefordert werden und stehen, wenn überhaupt, nur in Ballungszentren zur Verfügung [6, 17, 20]. Beim frankogermanischen Modell mit flächendeckendem Notarztwesen zeigt sich ein kontinuierlicher Anstieg von NA-Alarmierungen [13]. Mit Veröffentlichung der Benchmarkberichte des Teams des Ärztlichen Leiters Rettungsdienst (ÄLRD) Tirol wurde die hohe Rate an Notarzteinsatzfahrzeug(NEF)-Fehl- bzw. Übergabeeinsätzen vergleichbar gemacht [3].

## Hintergrund

Jeder Notfalleinsatz in Tirol beginnt mit einem Notruf bei der Leitstelle Tirol (LT). Seit September 2016 arbeitet diese mit dem Notrufabfragesystem NOAS (Fa. NOAS Notrufabfragesysteme GmbH, Egling), das auf standardisierten Abfrageprotokollen unter Einbindung des Notarztindikationskataloges der deutschen Bundesärztekammer (NIKDBÄK) basiert [1, 10]. Basierend auf der vom ÄLRD Tirol festgelegten Ausrückorder wird das entsprechende Rettungsmittel alarmiert [9].

**Figure 1:**
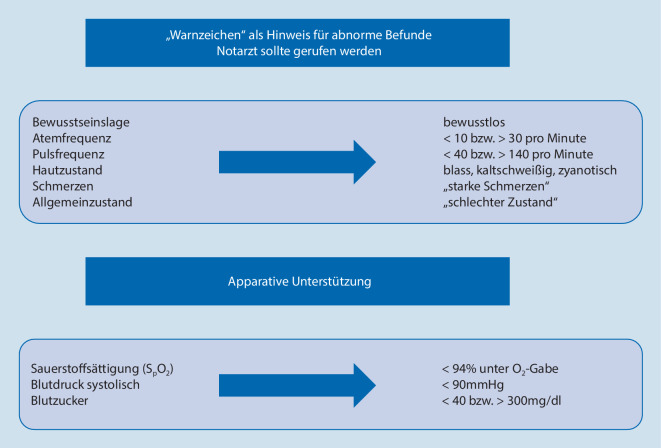

Sanitäter sollen einen Patienten anhand des ABCDE-Schemas beurteilen und sind nicht dazu ausgebildet, Diagnosen zu stellen [8, 14, 15]. Als Anhaltspunkt wurde eine Liste mit „Warnzeichen“ erstellt, jedoch ohne detaillierten Handlungsalgorithmus (Abb. 1; [11]).

Zum Tätigkeitsbereich von Rettungs- bzw. Notfallsanitätern gehören laut österr. Sanitätergesetz (SanG) 2002 die selbstständige und eigenverantwortliche Betreuung kranker und verletzter Personen vor und während des Transports sowie die Durchführung lebensrettender Sofortmaßnahmen. Bei NotfallpatientInnen („Notfallpatienten gemäß Abs. 1 Z 2 sind Patienten, bei denen im Rahmen einer akuten Erkrankung, einer Vergiftung oder eines Traumas eine lebensbedrohliche Störung einer vitalen Funktion eingetreten ist, einzutreten droht oder nicht sicher auszuschließen ist“ [5]) ist eine unverzügliche Anforderung eines Notarztes zu veranlassen. Die Sanitäter tragen für gesetzte oder unterlassene Maßnahmen die Verantwortung und haben eine Dokumentationspflicht. Sanitäter führen am Notfallort weder eine routinemäßige GCS-Einteilung noch eine NACA-Bewertung durch. Die Dokumentationspflicht im Rettungs- und Notarztwesen dient der Qualitätssicherung und der Nachvollziehbarkeit gesetzter Maßnahmen, u. a. auch zur Beweissicherung [8]. Die Einsatzdokumentation erfolgt in Tirol obligat für Notärzte und Rettungspersonal mit dem digitalen „Medical-Pad-CarPC“-Protokoll (CarPC MedicalPad® Version 7, TECH2GOMobile Systems GmbH, Hamburg); für Notärzte steht ergänzend und optional das handschriftliche NACA-X-Notarztprotokoll zur Verfügung [19].

Beim Rettungsdienstpersonal ist der länderspezifische Ausbildungsstandard zu unterscheiden. Die Ausbildung zum Rettungssanitäter umfasst in Österreich 260 h. Nach Abschluss kann die Ausbildung zum Notfallsanitäter mit weiteren 480 h erfolgen, die mit einem 40-stündigen Krankenhauspraktikum abschließt. Zusätzlich können allgemeine und spezielle Notfallkompetenzen erworben werden. Sehr verbreitet ist die Einbindung ehrenamtlicher Rettungs- und auch Notfallsanitäter [5].

Demgegenüber bedarf die Ausbildung zum Rettungssanitäter in Deutschland einer 3‑monatigen Vollzeitausbildung mit 520 h. Die Weiterbildung zum Notfallsanitäter dauert 3 Jahre und umfasst 4600 h. Somit unterscheidet sich der österreichische Notfallsanitäter deutlich vom deutschen Pendant insbesondere durch die wesentlich kürzere Ausbildungsdauer [21]. Die im angloamerikanischen Paramedic-System gebräuchliche Ausbildung zum „emergency medical technician – paramedic“ (EMT-P) dauert zwischen 2 und 4 Jahre. Hier wird unterschieden zwischen der Basisausbildung zum Emergency medical technician – basic (EMT-B) – entspricht etwa dem deutschen Rettungshelfer –, der weitergehenden Ausbildung zum Emergency medical technician – intermediate (EMT-I) – entspricht etwa dem deutschen Rettungssanitäter –, und der abschließenden Ausbildung zum EMT-P – entspricht etwa dem deutschen Notfallsanitäter. EMT‑P haben oft ein College-Studium abgeschlossen. Ein EMT‑P verfügt zumindest über folgende Kompetenzen: EKG-Interpretation, Medikamentengabe, erweitere Atemwegssicherung (z. B. „rapid sequence induction“), Reanimation von Polytraumapatienten sowie Kindernotfälle [6, 17, 20].

Die österreichische Notarztausbildung besteht aus einem 80-stündigen Notarztkurs sowie dem Absolvieren von 20 präklinischen notärztlichen Patientenversorgungen unter direkter Aufsicht eines verantwortlichen Notarztes und wird nach 33 Monaten klinischer Tätigkeit mit spezifischem Rasterzeugnis mit der Notarztprüfung abgeschlossen. Die Tätigkeit als Notarzt bleibt bis zur Erlangung der selbstständigen Berufsberechtigung auf klinikgebundene Systeme beschränkt [18].

Die Notarztausbildung in Deutschland setzt 24 Monate Weiterbildung in einem Gebiet der unmittelbaren Patientenversorgung in einem Krankenhaus, davon 6 Monate Weiterbildung in Intensivmedizin oder Anästhesiologie oder in der Notfallaufnahme an einer Weiterbildungsstätte voraus, woraufhin ein 80-stündiger Notarztkurs und 50 Einsätze unter Anleitung eines verantwortlichen Notarztes absolviert werden müssen [2].

## Studiendesign und Untersuchungsmethoden

### Datenerhebung

Die retrospektive Studie basiert auf den von der LT zur Verfügung gestellten Zahlen und Daten: 96.908 Rettungsdienstalarmierungen und 14.921 Notarztalarmierungen der Jahre 2017 und 2018 der Notarztsysteme NEF Innsbruck und Telfs, entsprechend einer Notarztquote von 15,4 % (Tab. 1). Unter Notarztalarmierungen fallen sämtliche NA-Ressourcetypen wie NEF, Notarzthubschrauber und niedergelassene Notärzte. Eine exakte Abgrenzung des Einsatzgebietes ist nicht zu treffen, da je nach Verfügbarkeit die Einsatzradien der NEF differieren; rettungsdienstliche und notärztliche Ressourcen unterstehen der nächsten Fahrzeugstrategie.

**Table 1:** 

	2017	2018	Summe
RD-Einsätze, gesamt	47.889	49.019	96.908
Einsätze mit NA (%)	7546 (15,8)	7375 (15,0)	14.921 (15,4)
Einsätze NA, primär (%)	6020 (12,6)	5843 (11,9)	11.863 (12,2)
Einsätze NA, Nachforderung (%)	1526 (3,2)	1532 (3,1)	3058 (3,2)
Einsätze ohne NA (%)	40.343 (84,2)	41.644 (85,0)	81.987 (84,6)

**Figure 2:**
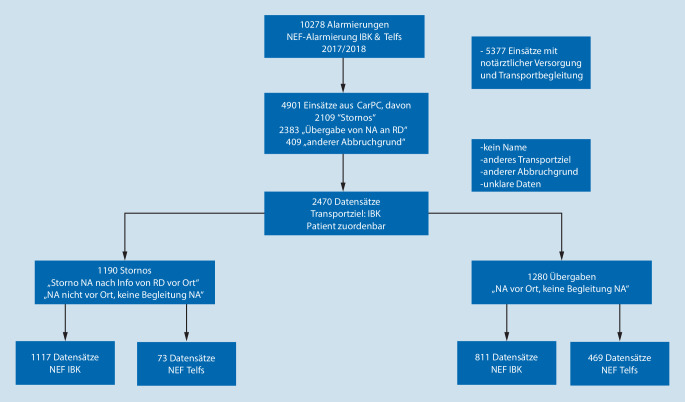

Aus dem CarPC wurden die Protokolldatensätze der Kategorien „Stornierung durch Rettungsdienst vor Ort“ und „Übergabe von Notarzt an Rettungsdienst“ gefiltert. Dem Krankenhausinformationssystem (KIS) des Landeskrankenhauses Innsbruck (LKI) wurden die Aufnahme- und Hauptdiagnosen der diesen Studiengruppen entsprechenden, stationär aufgenommenen Patientinnen und Patienten entnommen. Es ergaben sich 2470 auswertbare Datensätze, davon 1190 Storno- und 1280 Übergabeeinsätze. Zu der Kategorie „andere Abbruchgründe“ zählen die Angaben: „kein Patient vor Ort“, „böswillige Alarmierung“, „Ressourcentausch (anderes Notarztmittel schneller verfügbar)“ sowie „technische Probleme“. Unter „anderes Transportziel“ fallen alle Krankenhäuser, exklusive des Landeskrankenhauses/der Universitätsklinik Innsbruck (Abb. 2), des Tiroler Krankenhauses der Maximalversorgung. Patienten aus Innsbruck werden aufgrund der geografischen Lage am LKI versorgt; Patienten aus dem NEF-Gebiet Telfs werden auch in periphere Bezirkskrankenhäuser mit eingeschränkter Versorgungsmöglichkeit transportiert, sofern sie keine spezifischere Therapie benötigen. Dieser Studie steht aufgrund von datenschutzrechtlichen Gründen sowie des Votums der Ethikkommission nur der Datenzugriff für das LKI zur Verfügung. Der NEF-Stützpunkt Innsbruck wird ausschließlich durch Notärzte der Anästhesieklinik Innsbruck betrieben; das NEF-Telfs wird heterogen mit Ärzten mit gültigem Notarztdiplom und „ius practicandi“ besetzt und untersteht organisatorisch und qualitätssichernd der Univ.-Klinik für Anästhesie & Intensivmedizin Innsbruck. Weder in Innsbruck noch in Telfs bestehen für die eingeteilten Notärzte Parallelverpflichtungen.

Als Grundlage zur Beurteilung der gegebenen Notarztindikation wurde der Notarztindikationskatalog der Deutschen Bundesärztekammer (NIKDBÄK) 2013 verwendet [1]. Anhand dessen wurden die innerklinischen Aufnahme- und Hauptdiagnosen in „Stornierung/Übergabe gerechtfertigt“ und „Notarztindikation gegeben“ unter Miteinbeziehung des Patientenzustandes laut Sanitäterprotokoll/Notarztprotokoll eingeteilt. Die genaue Aufteilung, welche innerklinischen Diagnosen als „kritisch“ bzw. „stabil“ beurteilt wurden, befindet sich im Anhang. Stabile Vitalparameter, soweit dokumentiert, galten als Grundvoraussetzung für eine gerechtfertigte Stornierung/Übergabe. Folgende Grenzwerte wurden dazu definiert:

- Sauerstoffsättigung (S_p_O_2_) > 90 %,
- Herzfrequenz (HF) > 40/min und < 180/min,
- RR_sys_ > 90 mm Hg und < 180 mm Hg.

Aufgrund der oft lückenhaften Dokumentation galt ein Sanitäterprotokoll für diese Studie als nichtausgefüllt, wenn nicht einmal ein Vitalparameter oder das Notfallgeschehen dokumentiert wurde.

### Datenmengen

Leider differiert die Anzahl der Datensätze bei Übergabeeinsätzen aus CarPC und dem LT-Einsatzleitsystem (ELS) (2383 vs. 2695 – Abweichung 11,6 %), wofür mehrere Gründe bekannt sind:

- Es gibt Fälle, bei denen ein NEF alarmiert wurde, jedoch kein Protokoll zugeordnet wurde.
- NEF wird aus dem Einsatzleitsystem (ELS) mehrfach hintereinander zu einem Haupteinsatz alarmiert, dabei wird jedoch nur ein CarPC-Protokoll erzeugt.
- Falsche Stützpunktzuordnung der Ressource (sei es im ELS oder im CarPC).

### Einschluss‑/Ausschlusskriterien

Eingeschlossen wurden Patienten, bei denen es zwischen dem 01.01.2017 und dem 31.12.2018 laut CarPC zu einer Notarztstornierung bzw. einer Übergabe vom Notarzt an den RTW kam. Ausgeschlossen wurden Patienten, die nicht eindeutig mit Namen und Geburtsdatum zuordenbar waren, nicht ins LKI eingeliefert wurden bzw. zu denen kein Eintrag im KIS am Einsatzdatum gefunden wurde, ebenso wie unklare Stornierungen und Fehleinsätze.

### Statistik

Die Datenverarbeitung erfolgte mit der Software Microsoft Excel 2016 (Microsoft Corporation, Redmond, WA, USA), die statistische Auswertung mittels SPSS Version 26 (IBM, Armonk, NY, USA). 06:00 bis 18:00 Uhr wurde als Tag, 18:00 bis 06:00 Uhr als Nacht definiert. Patienten von 0 bis 17 Jahren wurden als Kinder definiert. Bei Folgeeinsätzen wurden 2 Zeitintervalle definiert: Folgeeinsatz innerhalb von 0–5 und 5–10 min nach Eintreffzeit.

Für die deskriptive Statistik wurde die prozentuelle Verteilung, für die statistische Signifikanztestung der T‑Test für unabhängige Stichproben für den Vergleich der Altersmittelwerte sowie der Chi-Quadrat-Test (χ^2^-Test) für Signifikanztestungen verwendet. Das Signifikanzniveau wurde mit *p* < 0,05 (signifikant) und *p* < 0,001 (höchstsignifikant) definiert.

## Ergebnisse

2470 Dokumentationen von 1263 versorgten Männern und 1207 Frauen wurden eingeschlossen und teilten sich in 1190 Stornierungen und 1280 Übergaben.

Am NEF Innsbruck fanden signifikant mehr Stornierungen statt als am NEF Telfs (15,4 % vs. 2,4 %; *p* < 0,05) und ebenso mehr Storno- als Übergabeeinsätze (*p* < 0,0001), umgekehrt am NEF Telfs mehr Übergabe- als Stornoeinsätze (*p* < 0,0001). Bei Kindern fanden signifikant mehr Storno- als Übergabeeinsätze statt (*p* < 0,05).

### Einfluss der Wochentage, Tageszeit und der Folgeeinsätze

**Figure 3:**
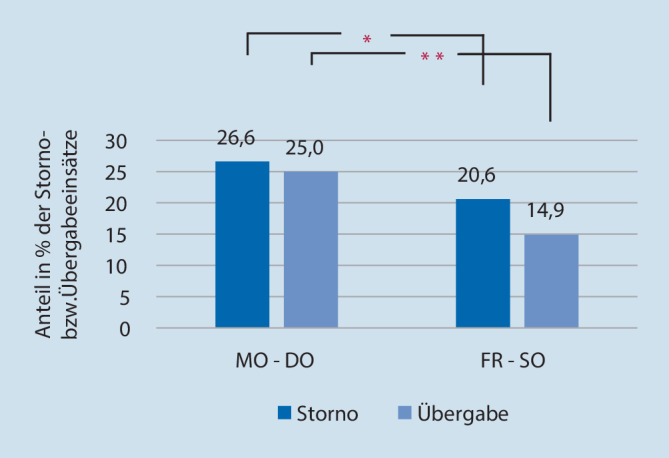

Nachts fanden an beiden NEF-Stützpunkten Freitag bis Sonntag weniger Storno- und Übergabeeinsätze statt als von Montag bis Donnerstag (*p* ≤ 0,05 (*) vs. *p* ≤ 0,0001 (**); Abb. 3).

Bei 40 (1,48 %) der 2695 dokumentierten Übergabeeinsätze wurde der Notarzt innerhalb von 10 min nach Eintreffen beim Patienten zu einem Folgeeinsatz alarmiert (Tab. 2).

**Table 2:** 

Folgeeinsätze während Übergabeeinsätzen	Zeitintervall
8 (0,3 % der Übergabeeinsätze)	0–5 min
32 (1,19 % der Übergabeeinsätze)	5–10 min

### Dokumentation im Sanitäterprotokoll

In 43,2 % der Storno- und 38,8 % der Übergabeeinsätze wurden keine Vitalwerte dokumentiert. In 23,9 % der Stornierungen und in 26,5 % der Übergaben war die Dokumentation der Vitalwerte und des Notfallgeschehens (Symptomatik, Verletzungsmuster etc.) der Sanitäterprotokolle unvollständig (Tab. 3).

**Table 3:** 

	Storno *n* (%)	Übergabe *n* (%)
Vitalwerte nicht dokumentiert	514 (43,2)	497 (38,8)
Gesamte Dokumentation unvollständig (kein Notfallgeschehen dokumentiert oder leeres Sanitäterprotokoll)	284 (23,9)	339 (26,5)
Sanitäterprotokoll ausgefüllt	906 (76,1)	941 (73,5)

### Notarztindikation

**Figure 4:**
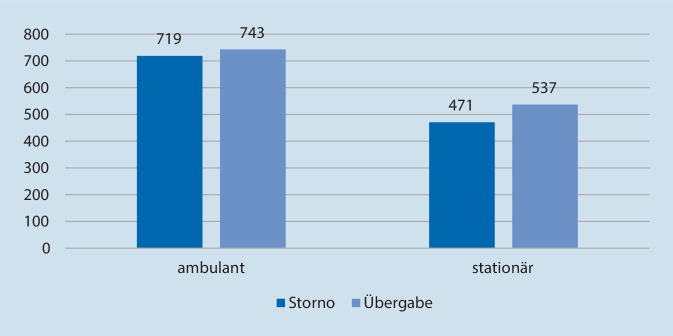

Bei 1462 (59,2 %) der 2470 eingeschlossenen und im LKI behandelten Patienten erfolgte eine ambulante Behandlung, bei 1008 (40,8 %) eine stationäre Aufnahme (Abb. 4).

Gemäß NIKDBÄK bestand retrospektiv in 210 Fällen (8,5 %) eine Notarztindikation, konkret bei 103 Stornierungen (8,7 % der Stornoeinsätze) und 107 Übergaben an den Rettungsdienst (8,4 % der Übergabeeinsätze). Patienten mit laut NIKDBÄK gegebener Notarztindikation mussten signifikant länger stationär behandelt werden (*p* < 0,05).

**Figure 5:**
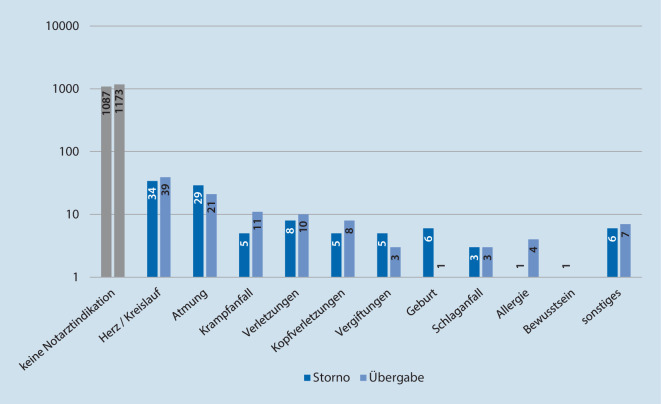

Die häufigsten Storno- und Übergabeeinsätze mit Notarztindikationen betrafen die Kategorien „Herz/Kreislauf“, „Atmung“, „Verletzungen“ sowie „Krampfanfall“ (Abb. 5).

### Kritische Vitalwerte

Bei der Beurteilung der Vitalparameter ergaben sich 127 kritische Vitalwerte, 45 betrafen Stornos und 82 Übergaben (Tab. 4).

**Table 4:** 

Vitalparameter	Storno	Übergabe
Blutdruck, systolisch < 90 mm Hg	10	16
Herzfrequenz < 40/min	3	1
Herzfrequenz > 180/min	1	3
Blutzucker < 40 mg/dl	1	4
Blutzucker > 300 mg/dl	2	1
Atemfrequenz < 10/min	0	1
Atemfrequenz > 30/min	4	2
Sauerstoffsättigung < 90 %	24	54
*Gesamt*	*45*	*82*

### Behandlung auf einer Intensivstation

35 Patienten (2,9 %) nach Storno- und 35 Patienten (2,7 %) nach Übergabeeinsätzen mussten auf einer Intensivstation aufgenommen werden, wobei retrospektiv nicht bei allen eine Notarztindikation gemäß NIKDBÄK gegeben war. Bei den Intensivbehandlungen nach einem Stornoeinsatz wurde bei 20 Patienten (1,7 % aller Stornoeinsätze) auch eine kritische Aufnahmediagnose festgestellt, nach den Übergabeeinsätzen bei 24 Patienten (1,9 % aller Übergabeeinsätze), somit besteht bei diesen ein Zusammenhang mit dem Notfallgeschehen.

## Diskussion

Die Ergebnisse zeigen, dass etwa jeder 5. Notarzteinsatz im Raum Innsbruck zu einer Stornierung des anfahrenden Notarztes sowie nahezu jeder 4. Einsatz zu einer Übergabe des Patienten an das Rettungsdienstpersonal führte. 1190 Stornierungen und 1280 Übergabeeinsätze aus 10.278 NEF-Alarmierungen wurden im Abgleich mit der innerklinischen Aufnahme- und Hauptdiagnose sowie den Sanitäterprotokollen anhand des NIKDBÄK untersucht.

Bei 210 Einsätzen (8,5 %) bestand ex post eine Notarztindikation – 103 Stornierungen (8,7 % der Stornoeinsätze) und 107 Übergaben an den Rettungsdienst (8,4 % der Übergabeeinsätze). Patienten mit laut NIKDBÄK gegebener Notarztindikation mussten signifikant länger stationär behandelt werden als Patienten ohne gegebene Notarztindikation.

Von Montag bis Freitag fanden nachts an beiden Stützpunkten weniger Storno- als auch Übergabeeinsätze statt. In knapp einem Viertel der Fälle (23,9 % der Storni und 26,5 % der Übergaben) lag im Sanitäterprotokoll keine Dokumentation vor.

### Tageszeiten und Transportwege

Am NEF Innsbruck kam es zu signifikant mehr Stornierungen und mehr Storno- als Übergabeeinsätzen als am NEF Telfs. Am NEF Telfs kam es zu mehr Übergabe- als Stornoeinsätzen. Diese Unterschiede könnten durch die geografische Lage erklärt werden. Beim NEF Telfs sind die Anfahrts- und Transportwege in die Klinik deutlich länger als in Innsbruck. Bei Patienten aus dem Bezirk Innsbruck-Land muss primär entschieden werden, ob ein Bezirkskrankenhaus wie z. B. Zams angefahren werden kann, oder ob ein Transport in eine Spezialabteilung der Universitätsklinik Innsbruck nötig ist. Vermutlich wird diese Entscheidung häufiger dem Notarzt überlassen und somit das Eintreffen des NEF abgewartet. Der ersteintreffende RTW muss in kurzer Zeit entscheiden, ob das anfahrende NEF gebraucht wird. Da sich ein Großteil der Notfallorte des Innsbrucker NEF/RTW in Kliniknähe befindet und in wenigen Minuten erreicht werden kann, wird ein anfahrendes NEF aufgrund des kurzen Transportwegs vom RTW eher storniert. Kritisch muss hinterfragt werden, ob die oft kurze Zeit bis zur Stornierung genügt, um ausreichende Informationen zu erhalten und die Vitalparameter zu erheben und zu beurteilen, oder ob die Stornierung zu voreilig getroffen wird bzw. die Sanitäter dabei nach dem „Load-and-go“-Prinzip handeln.

### Kinder

Dass es bei Einsätzen mit Kindern zu mehr Stornierungen als Übergabeeinsätzen kam, könnte dadurch erklärt werden, dass kindliche Notfälle gemäß der Tiroler Ausrückordnung vermehrt mit Notarztrettungsmitteln beschickt werden und daraus vor Ort dann öfter zu Notarztstornierungen führen; ist der Notarzt einmal vor Ort, wird er/sie das Kind potenziell inkl. der Eltern eher begleiten.

### Wochenende

An Wochenenden kam es an beiden Stützpunkten nachts zu weniger Storno- als auch Übergabeeinsätzen. Üblicherweise ist der Anteil an freiwilligen Rettungskräften an Wochenenden besonders hoch. Die geringere Stornorate könnte dadurch erklärt werden, dass ehrenamtliche Mitarbeiter weniger Erfahrung und Routine mitbringen und daher eine Stornierung eher unterlassen.

### Sanitäterprotokolle

Die Analyse der Sanitäterprotokolle diente als Hilfe, um Rückschlüsse auf den Patientenzustand vor Ort zu gewinnen. Evaluiert wurden Vitalwerte und Notfallgeschehen. Die Anforderung für die Bewertung des Notfallgeschehens war, dass mit den Angaben aus dem SanProtokoll zumindest ansatzweise Rückschlüsse auf das Notfallbild vor Ort gezogen werden konnten. Trotz dieser geringen Dokumentationsanforderungen war bei 23,9 % der Storni und 26,5 % der Übergaben die Dokumentation unvollständig.

Nach dem österr. SanG gehört die Dokumentation zu den Pflichten eines Sanitäters. Daher sollte auch von nichtärztlicher Seite darauf geachtet werden, dass diese Aufgabe ordnungsgemäß und mit voller Verantwortung durchgeführt wird [8].

Der Grundsatz „nicht protokolliert – nicht gemacht“ kann sich bei zivil- sowie strafrechtlichen Verfahren u. U. negativ auswirken. Eine Masterarbeit aus 2016 aus Zwickau, D, untersuchte die Dokumentationsqualität in der notfallmedizinischen Versorgung und kam zu einem ernüchternden Ergebnis: Lediglich 2,6 % aller Protokolle waren vollständig und variierten stark innerhalb von 9 verschiedenen ausgewerteten Kategorien bezüglich der Dokumentationsqualität [7].

### Notarztindikationen

Bei 103 Stornierungen sowie 107 Übergaben durch den Notarzt an den Rettungsdienst bestand nach dem Notarztindikationskatalog im Nachhinein eine Notarztindikation. Diese nichtgerechtfertigten Stornierungen bzw. Übergaben mussten signifikant länger stationär aufgenommen werden, 20 (1,7 %) Stornierungen und 24 (1,9 %) Übergabeeinsätze wurden mit einer kritischen Aufnahmediagnose auf eine Intensivstation aufgenommen. Dies zeigt, dass bei einem relevanten Anteil eine Notarztindikation sowohl bei Stornierungen als auch bei Übergabeeinsätzen bestand und somit eine schwerwiegende Erkrankung/Verletzung zugrunde lag und vom Sanitäter oder bei Übergabe an die Sanitäter vom Notarzt entweder noch nicht erkennbar oder falsch eingeschätzt wurde. Konsequenterweise fand auch eine längere Behandlung im Krankenhaus statt. Vergleichbare Literatur zu diesem Thema ist nicht zu finden.

Mehrere Studien evaluierten retrospektiv die Notarztindikationen anhand gesetzter Maßnahmen. Zum Teil ernüchternde Ergebnisse zeigten, dass ein Großteil der Alarmierungen keine spezifischen notärztlichen Maßnahmen erforderte und somit das gegenwärtige Modell der präklinischen Versorgung weder patientenorientiert noch effizient erscheint [4, 12, 13, 16]. Kritisch anzuführen ist hier, dass eine Notarztindikation definitiv nicht allein anhand gesetzter Maßnahmen zu beurteilen ist. Auch die Überwachung von potenziell lebensbedrohlich gefährdeten Patienten, ohne durchgeführte ärztliche Maßnahmen, kann einen Notarzt erfordern, ebenso wie Entscheidungen zur Belassung, soziale Notstände, Interhospitaltransporte, Transporte von niedergelassenen Ärzten mit bereits gesetzten Maßnahmen, polizeiliche Anforderungen bis hin zu psychologischen Hilfen/Atemanweisungen z. B. bei Hyperventilation etc. Weiters zu beachten ist, dass nicht nur im Ländervergleich, sondern auch regional unterschiedliche Rettungssysteme agieren. In Graz existiert seit Jahrzehnten ein 3‑stufiges System. Hierbei sind im Gegensatz zum 2‑stufigen System mit Rettungsdienstpersonal und Notärzten Jungärzte des „Medizinercorps“ mit hohem Ausbildungs- und Kompetenzgrad zwischengeschaltet [12]. Die Leitstelle Graz arbeitet mit dem freien Interview im Gegensatz zur LT, die mit NOAS arbeitet. Für die Steiermark gibt es einen von der Arbeitsgemeinschaft für Notfallmedizin Graz entworfenen Indikationskatalog zur Entsendung von Notarztmitteln [16].

### Folgeeinsätze

Bei 40 Übergabeeinsätzen wurde innerhalb von 10 min der Notarzt zu einem Folgeeinsatz alarmiert, sodass der Patient möglicherweise aus diesem Grund an den Rettungsdienst übergeben wurde.

### Limitationen

Kritisch anzuführen ist, dass nur 2 der 13 Tiroler NEF-Stützpunkte – je aber städtisches und ländliches Gebiet – in die Studie einbezogen und nur Patientinnen und Patienten mit Einlieferung ins Landeskrankenhaus Innsbruck inkludiert wurden. Eine weitere Limitation stellt der Patientenzustand am Notfallort dar; dieser kann sich rasch ändern und ist retrospektiv nur schwer beurteilbar. Ebenso limitierend ist die mangelhafte Dokumentation in Bezug auf eine objektive Auswertung des vorgefundenen Notfallgeschehens. Dabei lässt sich der Grund für die Stornierung des NEF bzw. für die Übergabe des Patienten an den RTW aufgrund mangelhafter Dokumentation nur schwer eruieren.

## Fazit für die Praxis

- Als Primärziel regen wir die Einführung eines standardisierten österreichischen Notarztindikationskataloges an. Damit könnten eine spezifischere Alarmierung durch die Leitstelle, die Nachvollziehbarkeit einer Belassung bzw. einer Übergabe vom NA an den RD sowie eine Hilfestellung für das Rettungsdienstpersonal erreicht werden und als Grundlage für weitere retrospektive Analysen zur Qualitätssicherung dienen.
- Der Dokumentationspflicht des Rettungsdienstpersonals sollte deutlich mehr Aufmerksamkeit geschenkt werden.
- Durch intensivere Aus- und Fortbildungen sowie Diagnosefeedbacks könnte die Anzahl an unberechtigten bzw. risikobehafteten Stornierungen/Übergabeeinsätzen vermindert werden.

## Supplementary Information




