# Supplementary material for: Evaluation of cancelled emergency physician missions and patient handovers in the area of Innsbruck: Retrospective assessment of physician-staffed emergency medical service cancellations and handovers from the emergency physician to the emergency medical service in 2017 and 2018
Source: Anaesthesist. 2021 Oct 13;71(4):272–80. [Article in German] doi: 10.1007/s00101-021-01046-y (PMC8986753; doi:10.1007/s00101-021-01046-y)
Supplement: Supplementary file 1 [file 101_2021_1046_MOESM1_ESM.pdf]

Zusatzmaterial zum Beitrag „Evaluierung der NEF-Fehl- und Übergabeeinsätze im Raum Innsbruck“ von Troppmair T., Egger J., Krösbacher A. et al. (2021) in *Der Anaesthesist.*

Beitrag und Zusatzmaterial stehen Ihnen auf www.springermedizin.de zur Verfügung. Bitte geben Sie dort den Beitragstitel in die Suche ein.

Auflistung der Notarztindikationen

Notarztindikation gegeben	Stornierung / Übergabe gerechtfertigt
**Kardial kritisch**<br>• Herzinfarkt (STEMI und NSTEMI)<br>• Hypertensive Krise (> 180 mmHg systolisch mit Symptomen)<br>• Tachykardie > 180/min<br>• Schock durch implantierten Defibrillator<br>• Instabile kardiale Dekompensation<br>• Kardiogener Schock<br>• Schrittmacherprobleme – kardial instabil<br>• Herzrhythmusstörungen – instabil<br>• Aortendissektion<br>• Lungenembolie<br>• Instabile Angina pectoris	**Kardial stabil**<br>• Endokarditis / Myokarditis – vital stabil<br>• Chronische Herzinsuffizienz<br>• Kardiale Dekompensation – vital stabil<br>• Herzrhythmusstörungen – vital stabil (Vorhofflimmern, …)<br>• Stabile Angina pectoris
**Atemnot kritisch**<br>• Atemnot bei COPD, Grad 3/4 akute Exazerbation<br>• Akuter Asthmaanfall / akute Exazerbation<br>• Status asthmaticus<br>• Pseudo-Krupp<br>• Epiglottitis<br>• Akute Laryngitis mit Atemnot bei Kindern<br>• Lungenödem/dekompensierte Herzinsuffizienz (vital nicht stabil)<br>• Spontanpneumothorax<br>• Akute Aspiration / akutes Bolusgeschehen<br>• Pleuraerguss mit Beeinträchtigung der Atmung	**Atemnot stabil**<br>• Akute Bronchitis<br>• Akute Laryngitis ohne Atemnot<br>• Pneumonie<br>• Oberer Atemwegsinfekt<br>• Hyperventilation<br><br>+ zusätzlich erfolgreiche Combivent-Anwendung bei COPD / Asthma durch NFS

Verletzung kritisch

- Polytrauma
- Offene Bauchverletzung
- Offene Brustkorbverletzung
- Pneumothorax (traumatisch bedingt)
- Starke verletzungsbedingte Schmerzen
- Wirbelsäulenverletzungen mit neurolog. Ausfällen
- Amputationen / Teilamputationen
- Großflächige Verbrennungen
- Innere Blutungen (Milzruptur, Leberruptur, Verletzung von Gefäßen, …)

Verletzung stabil

- Frakturen der Extremitäten ohne starke Schmerzen
- Diverse Riss-Quetsch-Wunden (RQW)
- Diverse Prellungen / Verstauchungen
- Luxationen ohne starke Schmerzen
- Blutungen ohne Schocksymptomatik
- Kleinflächige Verbrennungen bzw. Verbrennungen Grad I
- Komplikationen durch Endoprothesen

Schädelhirntrauma kritisch

- SHT Grad 2 und 3
- Subdural Hämatom traumatisch
- Epidural Hämatom traumatisch
- Cerebrale Kontusionsblutung

Kopfverletzungen stabil

- Commotio cerebri
- Oberflächliche Kopfverletzungen wie Riss-Quetsch-Wunden o.ä.
- Schädelbasisbruch (ohne kritische Begleitverletzungen)

Geburtshilfliche Notfälle

- Hausgeburt / Geburt im RTW
- Baby bereits sichtbar bzw. Wehenabstand < 2 Min.
- Presswehen
- Risikoschwangerschaft

Geburtshilfliche Notfälle

- Wehenabstand > 2 Minuten
- Keine Risikoschwangerschaft
- Nur Fruchtwasserabgang ohne Wehentätigkeit

Geburtshilfliche Notfälle NEF-pflichtig

- Hausgeburt / Geburt im RTW
- Baby bereits sichtbar bzw. Wehenabstand < 2 Minuten
- Presswehen
- Risikoschwangerschaft

Geburtshilfliche Notfälle ohne NA

- Wehenabstand > 2 Minuten
- Keine Risikoschwangerschaft
- Nur Fruchtwasserabgang ohne Wehentätigkeit

Krampfanfall kritisch

- Status epilepticus
- Andauernde bzw. rezidivierende Fieberkrämpfe
- Krampfanfall mit Bewusstseinsstörungen
- Grand-mal Anfall

Krampfanfall stabil

- Z.n. Krampfanfall, vital stabil
- Psychogene Krampfanfälle
- Patient wach/orientiert bei Eintreffen RD

Apoplex kritisch

- Apoplex mit Bewusstseinsstörung
- Hämorrhagischer Insult mit Bewusstseinsstörung

Apoplex stabil

- TIA (transitorische ischämische Attacke)
- Apoplex

Anaphylaxie kritisch

- Anaphylaktischer Schock

Anaphylaxie stabil

- Anaphylaxie stabil (z.B. nur Rötung bzw. Quaddeln ohne Atembeschwerden)
- Medikamentenreaktionen wie Steven-Johnson-Syndrom

Bewusstseinsstörungen kritisch

- soporöse Patienten
- Bewusstlosigkeit

Bewusstseinsstörungen stabil

- Somnolente Patienten
- Synkope

Intoxikationen kritisch

- Opiat / Benzodiazepine mit Bewusstseinsstörung
- Andere Intoxikationen mit Bewusstseinstrübung

Intoxikationen stabil

- Intoxikationen mit Alkohol (Patient ansprechbar)
- Andere Intoxikationen ohne Bewusstseinsstörung

Sonstiges kritisch

- Tumorkomplikationen vital nicht stabil
- Ertrinkungsnotfälle
- Schocksymptomatik (jegliche Ursache) wenn Vitalwerte schockentsprechend
- OP-Komplikationen mit hohem Blutverlust / Schocksymptomatik
- Hypoglykämie
- Starke Schmerzen bzw. Koliken (Patient ohne Schmerztherapie nicht transportfähig)
- drohender Suizid
- Meningitis / Encephalitis mit Bewusstseinsstörung
- Stromunfälle / Blitzschlag

Sonstiges stabil

- Tumorkomplikationen / tumorbedingte Symptomatik vital stabil
- Gastroenteritis (Übelkeit/Erbrechen)
- Reflux
- Fieberhafter Infekt
- Schwindel – div. Ursachen
- Hyperglykämie
- Psychiatrische Erkrankungen (exkl. drohender Suizid)
- Unwohlsein / Ermüdung
- Div. Schmerzsymptomatik (Pat. transportfähig)
- Div. Infektionen (z.B. Harnwegsinfekt, Wundinfektionen, …)
- Gynäkologische Notfälle (ohne Schwangerschaft)
- Nierensteine (wenn keine Schmerztherapie nötig)
- Niereninsuffizienz
- Chronische Pankreatitis
- Leberzirrhose
- Magenulcus
- Exsikkose (vital stabil)
- Migräne
- Akutes Abdomen
- Verschiedene Elektrolytentgleisungen
- OP-Komplikationen vital stabil
- Fremdkörper in Ösophagus (ohne Atemnot)
- Div. zahnärztliche Notfälle
  - Prinzipiell gerechtfertigt, wenn Vitalwerte und Patientenzustand stabil

---
